# A case of symptomatic double coronary artery-pulmonary artery fistulae

**DOI:** 10.4103/0974-2069.74050

**Published:** 2010

**Authors:** Suresh V Patted, Prabhu C Halkati, MD Dixit, Ravikant Patil

**Affiliations:** Department of Cardiology, KLE University, Belgaum, Karnataka, India

**Keywords:** Double coronary arteriovenous fistulae, pulmonary artery, surgical closure

## Abstract

A 57-year-old lady presenting with angina was found to have multiple coronary arterio venous fistulae (CAVF) arising from both left and the right coronary arteries and draining into the pulmonary artery. She underwent successful surgical closure of these CAVF.

## INTRODUCTION

Coronary arterio-venous fistula (CAVF) is an uncommon coronary anomaly reported in 0.002% of the general population. It was first described by Krause in 1865. The majority of CAVF are congenital in origin, representing 0.4% of all cardiac malformations. These abnormal connections can cause a hemodynamically significant left to right shunt and on rare occasions can affect myocardial perfusion.[1] Potential complications associated with CAVF include heart failure, myocardial ischemia, infective endocarditis, arrhythmias, and rupture. Many CAVFs, however, are asymptomatic and found incidentally. Our patient presented with symptoms mimicking ischemic heart disease (IHD) and had multiple CAVFs arising from both right and left coronary arteries and draining into the pulmonary artery.

## CASE REPORT

A 57-year-old lady presented with exertional dyspnea and NYHA class II angina since last 3 years which had aggravated in last 6 months. She had been previously diagnosed with hypertension and hypercholesterolemia and was put on medical treatment.

**Figure 1:**
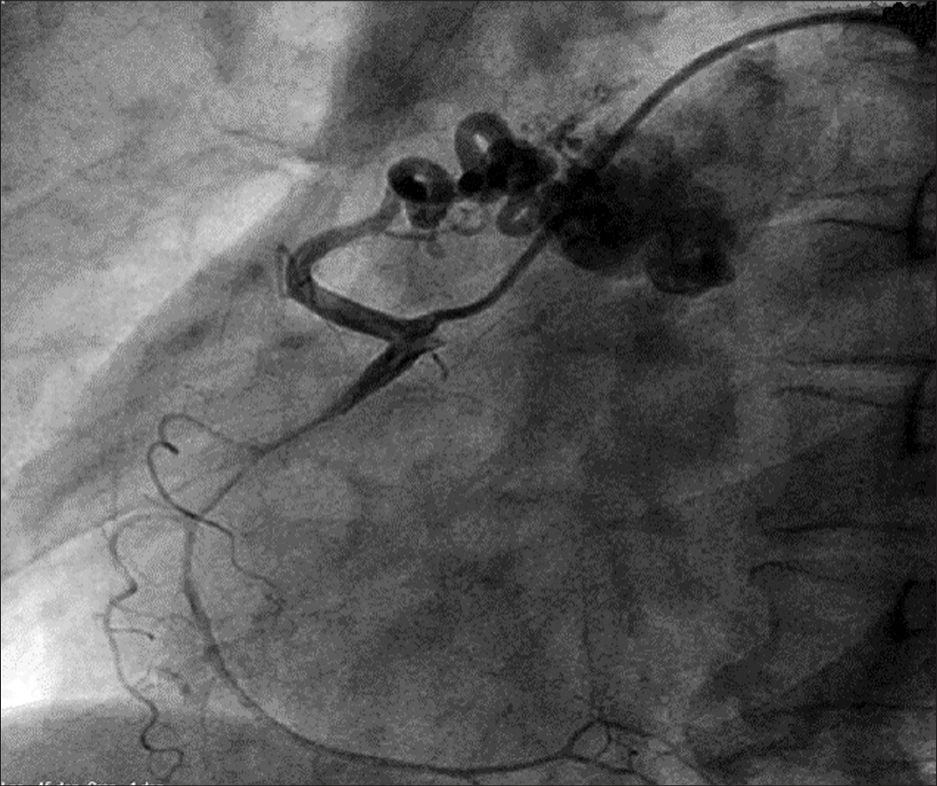
CAVF draining from RCA to PA

**Figure 2:**
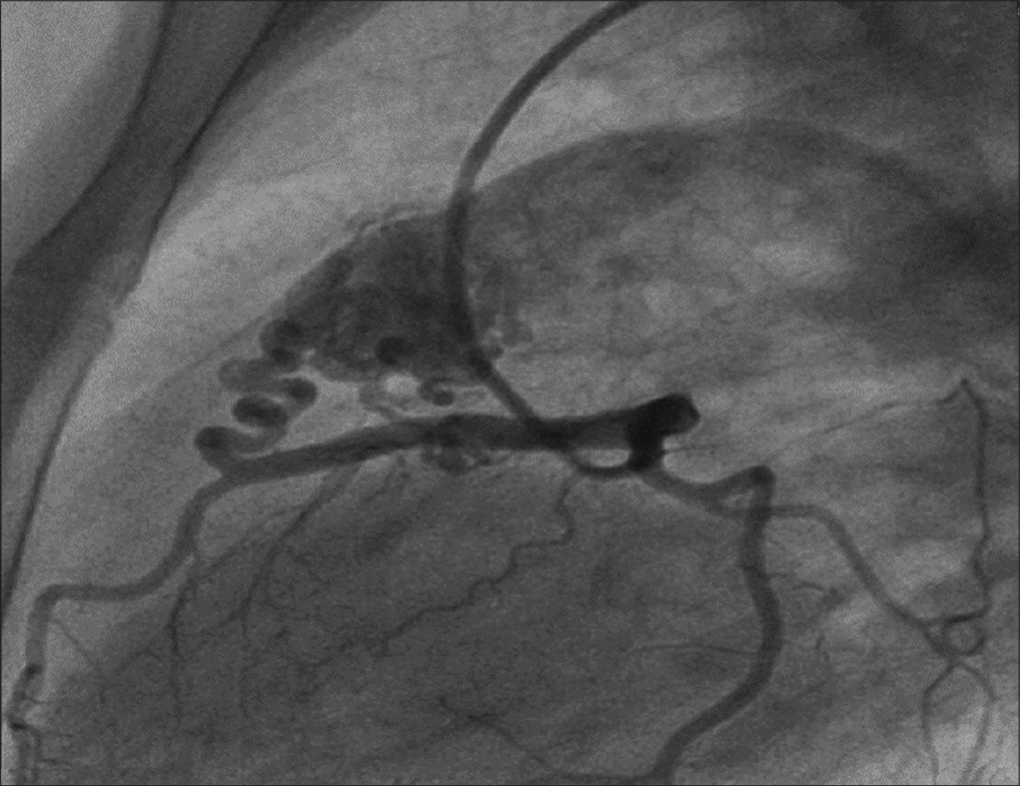
CAVF draining from LAD to PA

**Figure 3:**
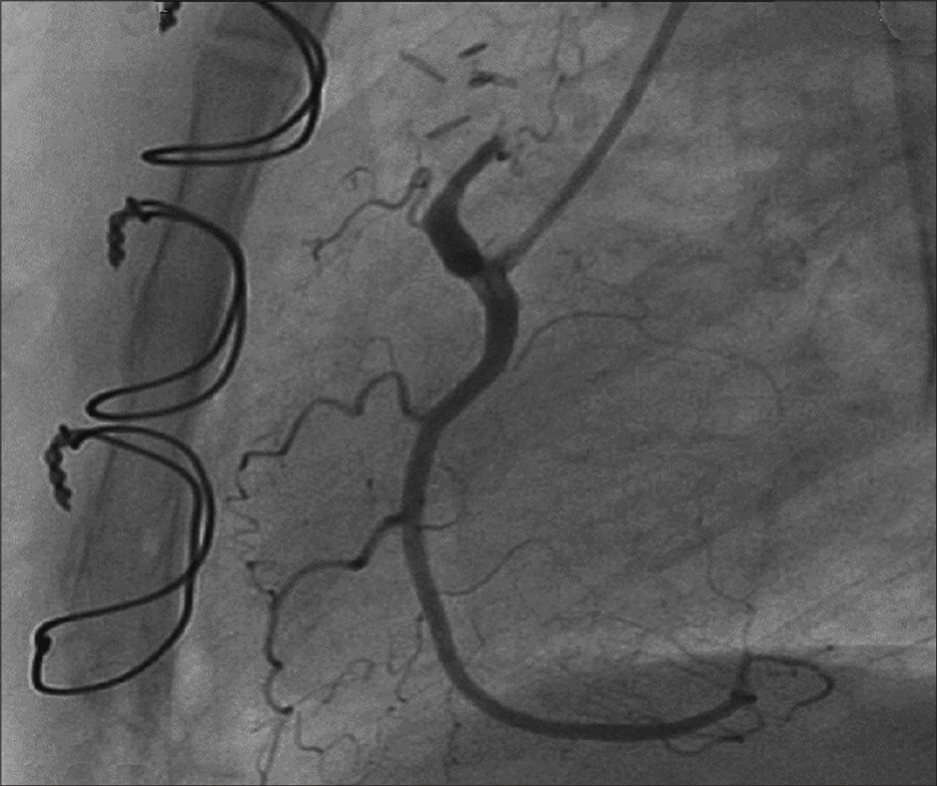
After surgical closure of CAVF draining from RCA to PA

**Figure 4:**
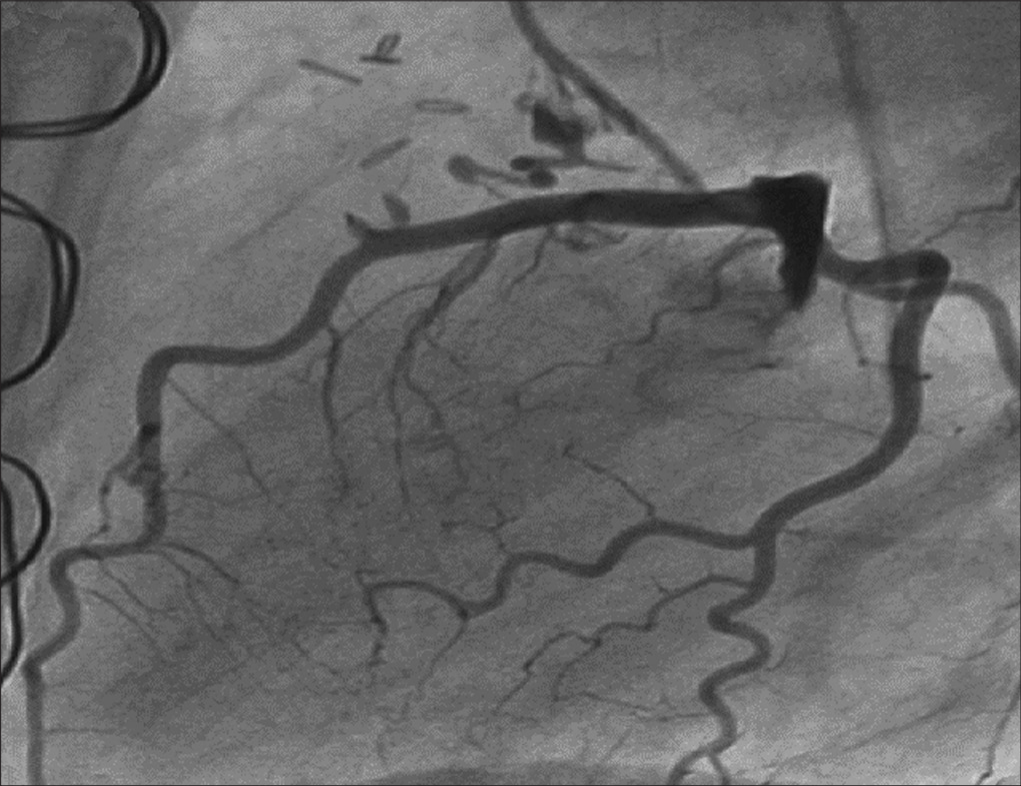
After surgical closure of CAVF draining from LAD to PA

Physical examination was unremarkable except a soft (grade II/VI) continuous murmur that was heard most clearly over the pulmonary area. An electrocardiogram was normal without any evidence of ischemia, and echocardiogram showed mild dilatation of the left atrium and left ventricle. Coronary angiography demonstrated multiple coronary artery to pulmonary artery fistulae originating from the left anterior descending (LAD) and right coronary arteries (RCA). There were no flow-limiting lesions in the coronary arteries. There was TIMI III flow in coronary vessels distal to the origin of the fistulae [Figures 1 and 2]. After a thorough discussion during the weekly catheterization meeting, it was decided to close the fistulae surgically in order to alleviate symptoms. Issues that prompted against transcatheter intervention were acute angle of origin of the fistula arising from LAD and the excessive tortuosity that would make transcatheter coil embolization of the coronary fistula extremely cumbersome and complex. Intra-operatively, the coronary fistulae were confirmed to be originating from the RCA and the LAD and terminating into pulmonary artery with an intervening “fistula lake.” The “lake” next to the LAD was identified, and the vessels feeding this lake were occluded with surgical clips as were the vessels coming from RCA. The aneurysmal sac was further obliterated using a larger surgical clip. Post-operative coronary angiogram showed no evidence of residual fistulae [Figures 3 and 4]. The patient made an uneventful recovery and at 1-month follow-up, she was symptom free.

## DISCUSSION

Congenital CAVF are the most common type of hemodynamically significant congenital coronary anomaly.[2] Nomenclature is based on a descriptive analysis of the vessel of origin and the chamber of termination e.g. LAD to right ventricular fistula. Angiographically the CAVF are broadly classified into two categories viz. Type A and B. Type A is a proximal type with the proximal coronary segment showing dilatation till the origin of the fistula while the distal end is normal, and Type B is the distal type wherein coronary artery is dilated over its entire length, terminating as a fistula in the right side of the heart (end-artery type). The coronary branching pattern proximal to the fistula is usually normal;[3] however, the distal branches are difficult to visualize in view of brisk run off from the fistula. Balloon occlusion angiograms are extremely helpful in defining the branching pattern of the coronary artery giving rise to a large fistula. Clinical symptoms associated with CAVF are variable and largely depend on the magnitude of the left-to-right shunt. In a large series of 51 patients with CAVF, angina pectoris occurred in 57% of cases as was seen in our patient. Moreover, angina has been found to occur in the absence of underlying coronary artery disease as was evident in this case.[4] Coronary “steal phenomenon” is the probable cause of angina in this subset of patients. The mechanism is related to the diastolic pressure gradient and runoff from the high-pressure coronary vasculature to a low resistance-receiving chamber or artery (e.g. pulmonary artery). Eventually, myocardium beyond the site of the fistula’s origin is predisposed to increased risk of ischemia, which is most frequently evident in association with increased myocardial oxygen demand during exercise or activity.

There is a general agreement that symptomatic patients with CAVF should be treated. This prompted us to offer our patient some form of therapy. The surgical obliteration of the fistula was the most preferred method until recently. However, with development in interventional techniques, percutaneous closure of fistula is feasible and in many cases preferable. Treatment modalities include coil embolization, use of Amplatzer vascular plug or PTFE-coated covered stent deployment.[5] Anatomy of the fistula and associated anomalies determine the type of intervention.[6] In our case, the presence of an aneurysm, two separate fistulous tracts, acute angulation of the origin of LAD fistula, and the excessive tortuosity of the proximal vessel prompted us to adopt the surgical technique.

Our case is a good example of how rare congenital coronary abnormality can remain completely silent for over decades and can very closely mimic the common diagnosis of IHD. Even though rare, diagnosis of CAVF should be kept in mind in all those who present with exertional angina or dyspnea as was the case in our patient.
